# Circulating Brain-Related miRNAs as Predictors of Postoperative Delirium in Cardiac Surgery Patients †

**DOI:** 10.3390/ijms26189062

**Published:** 2025-09-17

**Authors:** Karina Nowakowska, Grzegorz Walkiewicz, Emilia Stec-Martyna, Dominika Kulczycka-Wojdala, Emilia Frankowska, Piotr Miler, Agnieszka Pawlak, Katarzyna Woźniak, Michał Krejca, Mirosław Wilczyński, Jakub Kazmierski

**Affiliations:** 1 Department of Old Age Psychiatry and Psychotic Disorders, Medical University of Lodz, 92-216 Lodz, Poland; karina.nowakowska@umed.lodz.pl (K.N.); emilia.frankowska9107@gmail.com (E.F.); drpmiler@gmail.com (P.M.); 2 Department of Ophthalmology, Stanford University School of Medicine, Palo Alto, CA 94303, USA; gregory.walkiewicz@gmail.com; 3 Research Laboratory CoreLab, Medical University of Lodz, 92-215 Lodz, Poland; emilia.stec-martyna@umed.lodz.pl (E.S.-M.); dominika.kulczycka-wojdala@umed.lodz.pl (D.K.-W.); 4 Department of Affective and Psychotic Disorders, Medical University of Lodz, 92-216 Lodz, Poland; agnieszka.pawlak@umed.lodz.pl; 5 Department of Cardiac Surgery, Central Clinical Hospital, Medical University of Lodz, 92-213 Lodz, Poland; kaa.wozniak@gmail.com (K.W.); michal.krejca@umed.lodz.pl (M.K.); miroslaw.wilczynski@umed.lodz.pl (M.W.)

**Keywords:** miR-183-5p, delirium, oxidative stress, neuroinflammation, antioxidant activity

## Abstract

Delirium is a neuropsychiatric syndrome that is pathophysiologically related to both mental (dementia, depression) and physical illness. Its occurrence results in a poor prognosis. This study investigates whether specific miRNAs (miR-9-3p, miR-34c-5p, miR-96-5p, miR-183-5p, and miR-374-3p) related to brain function are associated with an increased risk of postoperative delirium. A total of 224 adult individuals scheduled for elective cardiac surgery were eligible to participate in the study. Delirium diagnosis was established with the use of the Confusion Assessment Method. Following miRNA expression profiling, cDNA synthesis was conducted on the samples obtained one day before and the day after surgery, from 60 delirium patients and 60 randomly selected non-delirium individuals. Univariate comparisons revealed that preoperative miR-96-5p (*p* = 0.05) and miR-183-5p (*p* = 0.001), along with postoperative miR-34c-5p (*p* = 0.009), miR-96-5p (*p* = 0.07), and miR-183-5p (*p* = 0.05), were associated with the risk of post-surgery delirium. However, after conducting multivariate logistic regression analysis, only miR-183-5p was found to be independently associated with the risk of delirium development. Other predictors of delirium included an ongoing episode of depression, peripheral vascular disease, female gender, active smoking, and increased postoperative pCO2 concentration. The current study revealed that preoperatively decreased expression of miR-183-5p predicts delirium development after cardiac surgery.

## 1. Introduction

Delirium is a neuropsychiatric syndrome that is commonly observed after major surgery, particularly among elderly patients with physical and psychiatric comorbidities, as well as those with impaired cognitive status [1,2]. It is characterized by sudden onset, fluctuations in attention and cognitive deficits, reduced awareness, and altered psychomotor activity. The frequency of delirium in elderly hospitalized individuals is exceptionally high, with rates of up to 70% [3]. Similarly, patients hospitalized in intensive care units due to COVID-19 infection have comparable rates of delirium at 73% [4]. Unfortunately, delirium is associated with poor prognosis, increased mortality, and impaired functioning even after the syndrome resolves [5].

The risk factors for delirium are well-known and described. However, investigations into its pathophysiology have only recently begun. The processes underlying delirium include an imbalance in the hypothalamic–pituitary–adrenal (HPA) axis, increased oxidative stress, and inflammation [6,7]. Despite significant progress, the role of miRNA in the development of delirium remains unclear.

MicroRNAs (miRNAs) are small, single-stranded RNA molecules that play a crucial role in post-transcriptional gene expression. Their post-transcriptional activity includes both the inducement of mRNA degradation and the stimulation of gene expression [8,9]. Specific miRNAs are recognized as regulators of three basic areas: central nervous system (CNS) development, neuroinflammation, and neurodegeneration.

The role of miRNAs in neural development was first demonstrated through conditional knockout mice experiments involving enzymes responsible for miRNA biogenesis [9]. For instance, miR-9 is highly expressed in neural precursors and regulates the number of neural stem cells [10,11]. Its overexpression controls proliferation and promotes neural differentiation by suppressing the orphan receptor [12].

Fu et al. (2020) conducted a study that demonstrated that miR-34c-5p is significantly reduced in individuals with drug-resistant epilepsy as compared to the control group [13]. The findings of the study suggest that the decrease in miR-34c-5p levels in drug-resistant epilepsy is responsible for inducing neuroinflammation, leading to the loss of hippocampal neurons and worsening of the disease. Additionally, Tu and Hu’s (2021) research found that the lowered expression of miR-34c-5p is associated with cerebral ischemia/reperfusion injury in experimental artery occlusion ischemic models and reperfusion-induced models in animal subjects [14]. The authors suggest that inflammatory and apoptotic signaling pathways may mediate the above process.

A recent study revealed that the upregulation of miR-96-5p increases the level of the glutamate transport-associated protein (GTRAP3-18), leading to a decrease in the concentration of the excitatory amino acid transporter (EAAC1) [15]. EAAC1 regulates the concentration of neuronal glutathione (GSH), an important neuroprotective antioxidant. When the level of EAAC1 decreases, GSH activity also diminishes, resulting in oxidative stress, neuronal damage, and the onset of neurodegenerative processes [15].

Although some miRNAs have been identified as regulators of the CNS and mediators of neuroinflammation, only a few are critical for regulating synaptic plasticity and cognitive status. One of these miRNAs is miR-9-3p, which is highly expressed in developing and mature brains [16]. Both miR-9-3p and miR-9-5p are produced from the miR-9 precursor and are involved in developing and losing neurons, synapses, and microglia activation [16,17]. The level of miR-9-3p is decreased in neurodegenerative diseases such as Huntington’s and Alzheimer’s disease (AD) [18,19]. It has been described as a biomarker differentiating between Parkinson’s disease (PD) and Multiple System Atrophy (MSA) [20].

Das Gupta et al. (2021) found that after experimental traumatic brain injury (TBI), plasma miR-9-3p levels increase [21]. The study showed that in patients with severe TBI, plasma miR-9-3p levels were 6.5- and 9.2-fold higher compared to those with mild TBI and the control group, respectively. These findings illuminate miRNA’s role in neuronal loss and brain tissue repair. In the study conducted by Wang et al. (2020), it was found that miRNA-183-5p expression in the mouse brain decreased after experimental intracerebral hemorrhage (ICH) [22]. The authors investigated the effect of miRNA-183-5p on the injury and repair of brain tissue after ICH by injecting miRNA-183-5p agomir or miRNA-183-5p antagomir into the lateral ventricles of mice with experimentally induced ICH. After several days of treatment, mice treated with exogenous miRNA-183-5p showed less brain edema, neurobehavioral defects, inflammation, oxidative stress, and ferrous deposition than antagomir-treated mice. Two studies have identified the specific miRNA involved in the development of postoperative delirium [23,24]. The first study found a correlation between microRNA-320 and postoperative delirium in patients undergoing tibial fracture internal fixation surgery [23]. The second study revealed that preoperative miR-210 could potentially predict the occurrence of postoperative delirium in elderly gastric cancer patients undergoing curative resection [24]. Recent research has shown that certain miRNAs can target genes that regulate the permeability of the blood–brain barrier (BBB) in both animal and human in vitro models. This discovery suggests that miRNAs have a role in modifying the integrity of the BBB. For instance, Coxsackievirus A16 can penetrate the BBB and decrease the expression of miR-1303 [25]. As a result, junctional complexes are disrupted by directly regulating matrix metallopeptidase 9 (MMP9), ultimately leading to pathological changes in the CNS [25]. The purpose of the present study was to investigate whether specific miRNAs (miR-9-3p, miR-34c-5p, miR-96-5p, miR-183-5p, and miR-374-3p) related to brain function are associated with an increased risk of delirium among patients who have undergone cardiac surgery.

## 2. Results

**Figure 1:**
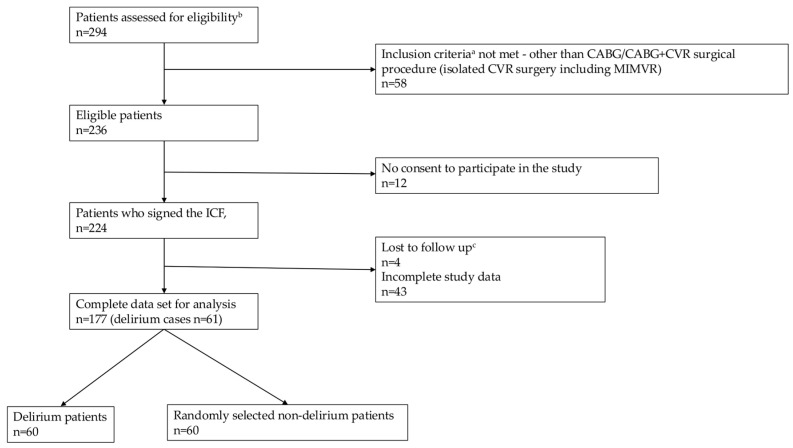
The selection process of eligible participants. CABG: coronary-artery bypass graft; CVR: cardiac valve replacement; MIMVR: Minimally Invasive Mitral Valve Repair. ^a^ Inclusion criteria: patients scheduled for cardiac surgery CABG/CABG with valve surgery, age ≥ 18 years. ^b^ Exclusion criteria: unstable general condition of the patient, diagnosis of dementia before surgery, delirium diagnosed in the week preceding the procedure, surgery other than CABG or CABG with CVR, chronic inflammatory or autoimmune diseases, use of corticosteroids, cytokine/anticytokine treatment 6 months before surgery, active alcohol or other substance addiction (abstinence period shorter than 3 months), severely impaired hearing or vision, illiteracy. ^c^ Death before the observation period was completed.

Elective cardiac surgery was performed in 294 patients during the study period; of these, 58 subjects did not meet the inclusion criteria since they underwent something different than CABG/CABG plus CVR surgery (isolated CVR surgery without CABG, including minimally invasive mitral valve repair [n = 58]), and 12 individuals did not sign an informed consent form. Of the 224 patients who signed their informed consent and were enrolled, 4 patients were lost to follow-up since they died before the observational period was completed, and 43 individuals had incomplete study data (these patients were not included in the analysis due to failure during sample collection or inappropriate sample collection (coagulation), n = 28, and due to incomplete postoperative delirium evaluation, n = 15). The incidence of delirium among the 177 remaining patients was 34% (61 delirium cases). After the miRNA expression profiling, cDNA synthesis was performed in 60 delirium patients and 60 randomly selected non-delirium individuals (simple randomization using the computer random number generation program). Figure 1 outlines the selection process of eligible participants, including accurate inclusion and exclusion criteria. miRNA dqPCR analysis was performed in the serum samples obtained one day before and the day after surgery (see Section 4.4).

### 2.1. Patients’ Characteristics, Univariate and Multivariate Comparisons

Study population characteristics are presented in Table 1.

The median preoperative and postoperative miRNA expression levels and oxidative stress biomarker concentrations in the whole population are presented in Table 2.

The findings of the univariate analysis of demographics, comorbidities, and factors related to anesthesia and surgical procedures are shown in Table 3 and Table 4.

Univariate comparisons revealed that preoperative miR-96-5p (*p* = 0.05) and miR-183-5p (*p* = 0.001) and postoperative miR-34c-5p (*p* = 0.009), miR-96-5p (*p* = 0.07), and miR-183-5p (*p* = 0.05) were associated with the risk of post-surgery delirium. However, according to the results of multivariate logistic regression analysis, only preoperative miR-183-5p was independently associated with the risk of postoperative delirium development (Table 5). Other predictors of post-surgery delirium included an ongoing episode of depression, peripheral vascular disease, female gender, active smoking, and an increased postoperative pCO2 concentration. There were no statistical differences between men and women with regard to miR-183-5-p expression. The median level of preoperative miR-183-5p among men and women in the whole study population was 164.5 (QR: 24.9–279.5) and 63.1 (QR: 14.5–174.8), respectively (*p* = 0.11, Cohen’s d coefficient = 0.29). The median level of postoperative miR-183-5p among men and women in the whole study population was 57.7 (QR: 5.5–181.0) and 47.7 (QR: 8.5–113.6), respectively (*p* = 0.53, Cohen’s d coefficient = 0.11). The median level of preoperative miR-183-5p among men and women in the delirium group was 51.5 (QR: 5.5–178.5) and 53.0 (QR: 17.9–166.3), respectively (*p* = 0.89, Cohen’s d coefficient = 0.03). The median level of postoperative miR-183-5p among men and women in the delirium group was 25.5 (QR: 0–93.2) and 55.3 (QR: 8.5–94.4), respectively (*p* = 0.36, Cohen’s d coefficient = 0.23).

### 2.2. Optimal miRNA Thresholds and Correlations with Other Analyzed Variables

According to the ROC analysis, the most optimal cutoff value of preoperative miR-183-5p that predicts the development of delirium was ≤193 copies/mL, with a sensitivity of 82%, specificity of 57%, positive predictive value of 0.65 and negative predictive value of 0.75 (area under the curve = 0.7; standard error = 0.05; 95% CI: 0.61 to 0.80; *p* < 0.001).

In the group of patients who did not experience delirium, there was a trend towards a significant correlation between preoperative miR-183-5p expression and preoperative superoxidase dismutase (SOD) activity (Spearman’s rank correlation 0.264; *p* = 0.09). However, a significant positive correlation was found between preoperative SOD activity and the preoperative levels of miR-96-5p (*p* < 0.05). Additionally, there was a significant positive correlation between postoperative miR-96-5p and preoperative plasma antioxidant activity, which is a marker that reflects the total activity of all plasma antioxidative agents (Spearman’s rank correlation 0.199; *p* = 0.04).

## 3. Discussion

The current study revealed that preoperatively decreased expression of miR-183-5p predicts delirium development after cardiac surgery.

In previous studies, upregulation of miR-183-5p has been reported to alleviate liver and brain injury induced by ischemia–reperfusion (I/R) [26]. Moreover, exosomal miR-183-5p was revealed to protect against myocardial I/R injury by targeting forkhead box protein O1 (FOXO1), reducing apoptosis and oxidative stress in I/R cardiomyocytes and improving cardiac function [27]. Zhu et al.’s study (2020) evaluated the effects of miR-183-5p on ischemia injury using ischemic models of mouse brains exposed to transient middle cerebral artery occlusion and Neuro-2A (N2A) neuroblastoma cells exposed to oxygen–glucose-deprivation (OGD) [26]. Their study investigated ischemia, miR-183-5p expression, N2A cell viability, and apoptosis-associated proteins’ expression. The results revealed that miR-183-5p expression was decreased, and brain damage was increased in ischemic mice compared with the sham group. Furthermore, N2A cells exposed to ischemia were characterized by lower miR-183-5p expression levels and increased apoptosis compared with the control group. Following the administration of agomiR-183-5p, cerebral ischemic injury and apoptosis were reduced in the stroke model and OGD-induced N2A cells.

Also, Wang et al. (2020) conducted a study to investigate the effect of miR-183-5p on brain injury in animal models [22]. Their analysis revealed that the expression of miR-183-5p decreased in the mouse brain after experimental intracerebral hemorrhage (ICH). However, mice treated with exogenous miRNA-183-5p showed improved brain edema, neurobehavioral defects, inflammation, oxidative stress, and ferrous deposition three days after ICH onset compared to control non-treated mice. As one of the miR-183-5p targets is heme oxygenase-1 (HO-1) (a molecule reported to exacerbate ICH brain injury), the authors investigated the association between agomir-183-5p and antagomir-183-5p injections, HO-1 levels, and cerebral injury. In the agomir group, the expression of HO-1 decreased significantly (*p* < 0.05), whereas there was no difference in its expression between the antagomir group and the ICH group. The study revealed that the positive impact of miR-183-5p on brain injury was related to inflammation and oxidative damage reduction via HO-1 inhibition.

Roser et al. investigated the role of microRNAs in Parkinson’s disease, hypothesizing that glial cell line-derived neurotrophic factor (GDNF) may enhance the survival of dopaminergic (DA) neurons in Parkinson’s disease (PD) models [28]. They demonstrated that transfecting synthetic miR-182-5p and miR-183-5p resulted in increased neurite outgrowth and provided neuroprotection for DA neurons both in vitro and in vivo, similar to the effects of GDNF. This effect was associated with decreased expression of the transcription factors FOXO3 and FOXO1 and enhanced PI3K-Akt signaling. Both of these transcription factors are known to promote neuronal apoptosis in response to oxidative stress and affect neurite growth [29].

In another study of animal models, extracellular vesicles derived from bone marrow mesenchymal stem cells carrying miR-183-5p were used to assess the impact on a diabetic intracerebral hemorrhage. The study showed that miR-183-5p alleviated neuroinflammation and oxidative stress via the PDCD4/NLRP3 pathway [30].

Interestingly, Zhou et al. revealed that extracellular vesicle-encapsulated miR-183-5p has a protective effect against the methamphetamine-induced dependence model in mouse brains by targeting neuregulin 1 [31]. Furthermore, in the prospective cohort study conducted among concussed children with and without persistent post-concussive symptoms (PPCS), expression of miR-183-5p was evaluated over time post-concussion. The results indicated statistically significant miRNA 183-5p overexpression after concussion in children with PPCS compared to children without PPCS [32].

**Figure 2:**
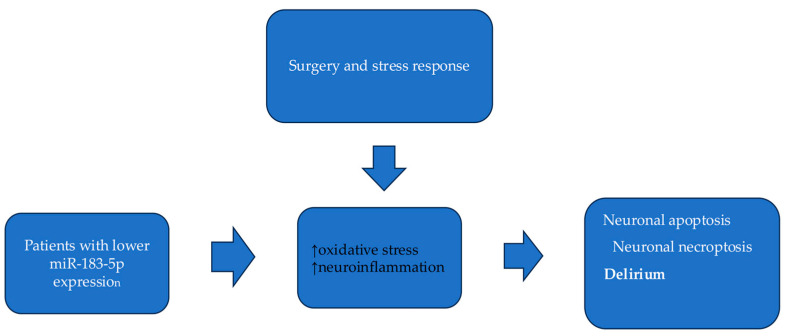
Proposed mechanism of miR-183-5p contributing to postoperative delirium. ↑: increase of process.

The present study is the first to reveal the role of miR-183-5p in the development of neurocognitive disorders. Specifically, the analysis showed that decreased miR-183-5p expression independently predisposes to postoperative delirium development among CVD patients. Available studies suggest that the mechanisms responsible for the impact of miR-183-5p on brain injury involve neuroinflammation and oxidative stress. As cardiac surgery burdens the brain’s functioning, lower expression of miR-183-5p may contribute to postoperative delirium via less efficient inhibition of neuroinflammatory and oxidative stress processes. Current evidence suggests that miRNAs are involved in pathways that regulate redox biology [33]. In their experimental study, Li et al. propose that miR-183-5p specifically serves as a protective factor in neuronal survival. MiR-183-5p reduces neuronal death by regulating two distinct cell death pathways: PDCD4 (a key protein in cell apoptosis) and RIPK3 (a regulator of necroptosis) [33]. Furthermore, their study indicates that miR-183-5p is stress-inducible, meaning it acts as an immediate response factor to neuronal stress. Therefore, miR-183-5p regulates stress sensing and response in neurons and is crucial for neuron survival under stress conditions. This is a potential pathogenic pathway in the current study, where patients with lower miR-183-5p expression are more vulnerable to oxidative stress, neuronal death and delirium [Figure 2].

Our previous studies revealed that CABG patients with decreased preoperative antioxidant activity (less efficient antioxidative mechanisms) and those with depressive episodes complicated with lower postoperative antioxidant activity are at significantly higher risk of delirium after cardiac surgery [6]. As pre- and postoperative antioxidant capacity levels were negatively correlated with postoperative soluble receptor for advanced glycation end products (sRAGE) concentration, we concluded that sRAGE overexpression may be a protective mechanism against increased oxidative stress and subsequent cell damage. In another study, we found that individuals with less efficient baseline antioxidative mechanisms have a higher postoperative peak of myeloperoxidase (MPO), a lysosomal enzyme known for its strong pro-oxidative and pro-inflammatory properties [2]. Consequently, these patients were more susceptible to experiencing delirium after surgery. Additionally, a higher preoperative plasma concentration of monocyte chemoattractant protein-1 (MCP-1)—a key chemokine involved in neuroinflammation and myelin degradation—has been identified as a predictor of postoperative delirium development [7]. Other studies among non-cardiac surgery patients also revealed the role of inflammation in postoperative delirium development [34,35].

The reason for decreased preoperative miR-183-5p expression in the studied population is unknown. However, the present analysis revealed that individuals with higher preoperative superoxide dismutase (SOD) concentration who did not develop delirium were characterized by increased miR-183-5p levels (a trend toward significance; *p* < 0.1). The same positive correlation was observed between preoperative SOD activity and miR-96-5p levels (*p* < 0.05). Additionally, a significant positive correlation was revealed between postoperative miR-96-5p and preoperative plasma antioxidant capacity (*p* < 0.05).

SOD is the first line of antioxidative defense, and its increased activity was associated with protective effects against mortality from cancer [36] and with lower all-cause mortality in older women [37]. Antioxidant capacity (AC) reflects the cumulative action of all antioxidants present in plasma, and its lower measures may indicate higher vulnerability to diseases related to oxidative stress.

Interestingly, in previous studies, increased miR-183-5p expression was significantly upregulated post-concussion in children with persistent post-concussive symptoms (PPCS) as compared to those without PPCS. The different distributions of the identified miRNAs in children with vs. without PPCS may signal a differential physiological response to the concussive injury or its subsequent repair [32].

Based on the mentioned findings, it can be hypothesized that specific miRNAs may have protective mechanisms against excessive oxidative stress and neuronal damage. The presence of miR-183-5p may indicate a curative role, while reduced levels of this miRNA could increase the risk of neuronal damage following cardiac surgery and other traumas. Notably, in univariate analysis, we identified additional associations, specifically between miR-34c-5p, miR-96-5p, and delirium. In Hamada et al.’s study, miR-34c-5p was significantly downregulated after ischemia, which was correlated with cognitive decline [38]. Also in our study, univariate analysis revealed lower postoperative expression of miR-34c-5p among patients with delirium. Based on animal studies, downregulation of miR34c-5p may contribute to delirium through hippocampal neuronal loss and increased neuroinflammation. A similar observation was made for miR-96-5p. Overexpression of miR-96-5p suppresses pyroptosis and reduces brain damage during the acute phase of ischemic stroke in an animal model, with the opposite effect observed when miR-96-5p expression decreases [39].

### Limitations

This well-designed and innovative study has some limitations. MiRNA was measured only once before the operation and once after; there were no additional tests to evaluate how miRNA expression changed over time postoperatively. However, in the current study, we aimed to assess the predictive power of miRNA in the development of delirium. Therefore, the postoperative sampling was carried out preoperatively and on the first postoperative day, before the onset of postoperative delirium. To our knowledge, there is only one study on a group of 30 people, of whom only 12 had delirium, in which miR was assessed at three time points (start of surgery, end of surgery, and 24 h after surgery) [40].

Additionally, the entire study group consisted of patients with advanced cardiovascular disease, and there was no control group without CVD for comparison. Recent studies have shown that patients with carotid atherosclerosis exhibit higher serum levels of miR-183-5p compared to healthy individuals [41,42]. Increased levels of circulating miR-183-5p were also detected in patients with acute coronary syndrome (ACS) and non-ST-segment elevation myocardial infarction (NSTEMI) [43,44], with the observation that serum miR-183-5p levels positively correlated with the Gensini score and hs-CRP [36]. Additionally, further investigations revealed a positive correlation between circulating miR-183-5p levels and coronary artery disease (CAD) compared to individuals without CAD [45]. According to the studies previously mentioned, miR-183-5p has a protective effect not only on neurons but also on cardiomyocytes. Our patient group consisted of individuals with CVD, making it challenging to determine whether the higher expression of miR-183-5p in those with a lower risk of delirium was linked to a cardioprotective or neuroprotective mechanism or a general effect against oxidative stress. This raises the question of whether the direct examination of miR-183-5p overexpression in neurons and cardiomyocytes should be pursued. On the other hand, our analysis revealed that only preoperative miR-183-5p was independently associated with postoperative delirium. It may suggest that the postoperative increase in miR-183-5p, related to the systemic stress response or change in CAD severity, has a lesser impact on the risk of delirium development. Furthermore, as described in the laboratory section, we initially assessed the expression levels of 754 human miRNA genes in serum samples from four patient groups: samples obtained before and after surgery from patients who developed delirium, and samples obtained before and after surgery from patients who did not develop delirium. Of note, only miRNAs with Cq  <  35 were considered detected, and miRNAs with altered expression profiles were selected for further investigations. Furthermore, the severity of CAD and heart failure, as well as other perioperative factors, were included in our multivariate analysis to ensure that the expression of miR independently distinguishes between delirium and non-delirium patients. Although the study included a large number of participants, only 120 (60 with delirium and 60 without delirium) were assessed in detail, which may be a limitation. However, we entered into analysis a number of variables associated with demography, mental and physical condition, and surgery. Therefore, the impact of different potential risk factors was analyzed in multivariate comparisons.

## 4. Materials and Methods

The study received approval from the Ethics Committee of the Medical University of Lodz, Poland, under Approval Number RNN/95/17/KE. The procedures followed ethical standards in accordance with the Declaration of Helsinki. Adult individuals scheduled for elective cardiac surgery between April 2017 and November 2019 were eligible to participate. Patients were included if they provided informed consent and were set to undergo isolated coronary artery bypass grafting (CABG) surgery or CABG with cardiac valve replacement (CVR). Participants experienced either on-pump (with cardiopulmonary bypass (CPB)) or off-pump surgery; however, the effects of CPB on the risk of postoperative delirium were controlled for in the statistical analysis. The exclusion criteria included concomitant surgeries other than CABG or CABG with CVR; preoperative delirium; active alcohol or other substance addiction (with an abstinence period shorter than three months); illiteracy; and significant hearing or visual impairment. Participants were recruited consecutively.

### 4.1. Neuropsychiatric Assessment

Patients’ cognitive status was assessed using the Mini-Mental State Examination (MMSE) the day before their scheduled operation [46]. Diagnostic criteria from the Diagnostic and Statistical Manual of Mental Disorders (DSM-5) were used to diagnose Major Depressive Disorder (MDD) and anxiety disorders [47]. Each patient was screened for delirium once daily using the Confusion Assessment Method for the Intensive Care Unit (CAM for ICU) and the Memorial Delirium Assessment Scale (MDAS), both of which evaluate the past 24 h [48,49]. After surgery, all patients were monitored in an intensive care unit, where they received specialized medical care and enhanced monitoring. If there were doubts or discrepancies in the CAM and MDAS assessments, the final consensus on delirium diagnosis was determined by the study team of physicians, who reviewed all available information. Nurses and doctors were interviewed, and clinical notes were examined for mentions of delirium or its symptoms.

### 4.2. Anesthesia

For the induction of anesthesia, fentanyl (5–10 mcg/kg), propofol (1–2.5 mg/kg), and rocuronium (0.6–1.0 mg/kg) were administered. During the maintenance phase, fentanyl was delivered via continuous intravenous infusion at doses of 2–10 mcg/kg/h, with propofol at 3–10 mg/kg/h and intermittent doses of rocuronium. Ventilation was provided with a breathing mixture of FiO_2_ 0.5 and air to maintain end-tidal CO_2_ levels between 35 and 45 mmHg. From the surgical incision to connection with cardiopulmonary bypass, sevoflurane (0.5–2 vol%) was used. After the surgical intervention, patients were transferred to the ICU for continued mechanical ventilation. Until extubation, morphine was administered via continuous infusion at a rate of 1–2 mg/h, alongside propofol perfusion at 1–2 mg/kg/h for sedation. The criteria for extubation included arterial blood gases and oxygen saturation greater than 92%, along with stabilization of hemodynamic parameters. Patients were included if they signed informed consent and presented for isolated CABG surgery or CABG surgery with cardiac valve replacement (CVR). Patients with CABG underwent both on-pump (without cardiopulmonary bypass (CPB)) and off-pump (with CPB) surgery; however, the impact of CPB on the risk of postoperative delirium was controlled in the statistical analysis. The exclusion criteria were as follows: concomitant surgery other than CABG or CABG with CVR; preoperative delirium; active alcohol or other substance addiction (abstinence period shorter than three months); illiteracy; pronounced hearing and/or visual impairment. The participants were recruited consecutively.

### 4.3. Surgery

Patients who underwent coronary artery bypass grafting (CABG) or CABG with concurrent valve surgery were operated on using median sternotomy and placed on cardiopulmonary bypass (CPB) under normothermic conditions. All patients received anterograde Del Nido cardioplegia during the procedure. In some cases, CABG was performed off-pump, meaning the surgery was conducted on a beating heart. This approach was carried out either through median sternotomy or through a left-sided mini-thoracotomy.

### 4.4. Laboratory Measurements

Venous blood samples were collected on two occasions during the study period: once the day before the surgery (baseline measurement) and again on the first postoperative day, between 7:00 and 9:00 a.m. The blood samples were centrifuged at 7000 rpm for 10 min and then frozen at −80 °C until the biochemical parameters were analyzed.

### 4.5. Exosome Isolation

According to the manufacturer’s instructions, total exosomes were extracted using the miRCURY Exosome Serum/Plasma Kit (Cat No. 76603; Qiagen, Hilden, Germany). In brief, plasma was thawed on ice and centrifuged at 3000× *g* for 10 min at 4 °C. Then, 800 µL of supernatant was transferred to a new tube, and 8 µL of thrombin (stock concentration of 500 U/mL) was added and incubated for 5 min at room temperature. The plasma was centrifuged at 10,000× *g* for 5 min, and 700 µL of the supernatant was transferred to a new tube. Then 280 µL Precipitation Buffer A was added, incubated for 60 min at 4 °C, and centrifuged twice at 500× *g* for 5 min to remove the supernatant. The pellet was resuspended in 240 µL of Resuspension Buffer for RNA extraction.

### 4.6. Isolation and Purification of miRNA

Total RNA, including miRNA, was extracted using the miRNeasy Mini Kit (Cat No. 217004; Qiagen, Germany) according to the manufacturer’s protocol with one minor variation. After the sample was mixed with 700 µL of QIAzol Lysis Reagent, 5 µL of 5 nM cel-miR-39-3p (Cat No. 4464066, Ambion, Austin, TX, USA) was added as an exogenous spike-in control. RNA was eluted from spin columns in 30 μL of RNase-free water and stored at −80 °C until use.

### 4.7. Expression Profile of miRNA Genes

Broad miRNA profiling was carried out using TaqMan^®^ Human MicroRNA Array A and B (Cat No. 4444913, Thermo Scientific, Waltham, MA, USA) as described by the manufacturer. Briefly, 3 μL of RNA extracts was reverse-transcribed using MegaplexTM RT Primers Human Pool A and B (Cat No. 4444745, Thermo Scientific) in a final volume of 7.5 μL. cDNA targets were then pre-amplified using 2.5 μL of the RT product in a 25 μL pre-amplification reaction. The PCR mixture for each array contained 450 μL of TaqMan Universal PCR Master Mix, 9 μL of diluted PreAmp product, and 441 μL of nuclease-free water. The plates were inoculated with 100 µL of the mixture into each port and sealed. Amplifications were performed using the 7900HT Fast Real-Time PCR System (Thermo Scientific) under the following conditions: 2 min at 50 °C, 10 min at 94.5 °C, and 40 cycles each for 30 s at 97 °C and 1 min at 57 °C. The expression levels of 754 human miRNA genes were assessed in serum samples of 4 patient groups: samples obtained before surgery from patients who developed delirium (n = 10), samples obtained after surgery from patients who developed delirium (n = 10), samples obtained before surgery from non-delirious patients (n = 10), and samples obtained after surgery from non-delirious patients (n = 10). Only miRNAs with Cq  <  35 were considered as detected. miRNAs with altered expression profiles were chosen for further investigations.

### 4.8. cDNA Synthesis and Digital Quantitative PCR

According to the manufacturer’s instructions, the purified total RNA was reversely transcribed into cDNA using the TaqManTM advanced miRNA cDNA Synthesis Kit (A28007, Thermo Scientific, Waltham, MA, USA). The cDNA synthesis protocol consisted of a poly(A) tailing reaction, a ligation reaction adding an adaptor sequence, and a reverse transcription reaction; 5 µL of RT product was pre-amplified to increase the amount of cDNA for all miRNAs (miR-Amp reaction) uniformly and stored at −20 °C. Selected miRNAs were profiled using digital quantitative PCR with a QX200 droplet digital PCR system (Bio-Rad, Warsaw, Poland) according to the manufacturer’s instructions. The ddPCR mixture was composed of 11 µL of 2 × ddPCR Supermix for Probes (No dUTP) (Bio-Rad), 1.1 µL of appropriate TaqMan Advanced miRNA Assay (hsa-miR-96-5p (Assay ID: 478215_mir), hsa-miR-34c-5p (Assay ID: 478052_mir), hsa-miR-9-3p (Assay ID: 4782151_mir), hsa-miR-183-5p (Assay ID: 477937_mir) or hsa-miR-374b-3p (Assay ID: 479421_mir)), 8.9 µL DNase/RNase free MilliQ water and 1 µL of 10 time-diluted cDNA, in a final reaction volume of 22 μL. A Bio-Rad QX200 droplet generator was used to partition each PCR reaction into up to 20,000 nano-sized droplets by loading 20 μL of the reaction mixture and 70 μL of droplet generation oil for probes (Bio-Rad) onto matched wells of a DG8 cartridge (Bio-Rad). Then, 40 μL of the droplet/oil mixture was transferred to a 96-well plate (Bio-Rad). The plate was then heat-sealed using a PX1 PCR plate sealer (Bio-Rad) set to run at 180 °C for 5 s. The PCR was performed in a T100 Thermal Cycler (Bio-Rad). The PCR reaction setups and thermal cycling conditions used for individual miRNA quantification are summarized in Appendix A, Table A1.

The fluorescence signals were measured by the QX200 Droplet Reader (Bio-Rad). The positive droplets containing amplified products were distinguished from negative droplets by applying a threshold above the negative droplets. Reactions with more than 10,000 accepted droplets per well were analyzed using QuantaSoft™ Analysis Pro software version 1.0.596 (Bio-Rad). Subsequently, the results were converted into copies/1 mL of the input material concerning the input plasma volume and dilutions at the RT and PCR reaction levels.

### 4.9. Statistical Analysis

Quantitative variables are expressed as medians and interquartile ranges (IQRs). For categorical variables, the number of observations (n) and fraction (%) were calculated. Normality was tested using Shapiro–Wilk’s test for normality. Differences between two independent samples for continuous data were analyzed using the Mann–Whitney U test (since the distributions of variables were different from normal). The effect size for continuous variables was calculated with Cohen’s d. For categorical variables, statistical analysis was based on the chi-squared test or Fisher’s exact test. Cramer’s V coefficient was calculated to assess the effect size for categorical variables. Spearman’s rank correlation coefficients were calculated to determine the correlation between two quantitative variables. The minimum study sample size was calculated using the power analysis, estimating the expected effects from our previous studies and assuming an alpha level of 0.10 and a power of 80% (the minimum sample size for each group is 37 patients). To evaluate the discriminant power of miRNAs, receiver operating characteristic (ROC) curves were drawn (Area Under Curve with Standard Error was given), and optimal decision thresholds (based on Youden’s index value) were found. The sensitivity, specificity, and positive and negative predictive values were calculated. Odds ratios with 95% confidence intervals were also presented. All investigated miRNAs and other factors significant in univariate comparisons (*p* < 0.10) were included in a forward stepwise logistic regression model to identify independent risk factors for delirium. The results were considered significant for *p* < 0.05. The calculations were performed using STATISTICA (version 13.3, 2017; StatSoft, Inc., Tulsa, OK, USA) and the R project (the rcompanion package, Version 2.4.15).

## 5. Conclusions

The current study indicates that patients with reduced preoperative expression of miR-183-5p face a greater risk of experiencing postoperative delirium. This association may be related to increased oxidative stress and neuroinflammation in this patient group.

The potential pathogenic pathway of this association may be related to increased oxidative stress and neuronal death, mediated by PDCD4 and RIPK3 regulators.

## Figures and Tables

**Table 1:** Demography, comorbidities, and perioperative characteristics of the study participants.

Characteristic	Mean or N	SD or %
Age (years)	68.13	6.80
Gender (Male)	92	76.67%
Weight (kg)	81.69	12.38
Height (cm)	170.22	8.20
Education (years)	11.74	3.75
CDT test	5.77	2.44
MMSE Score	27.44	2.22
Ejection fraction (%)	51.68	10.26
CCS score	2.47	0.77
NYHA grade	2.18	0.72
Urea concentration (mmol/L)	7.58	8.76
Creatinine concentration (μmol/L)	90.59	35.70
Presence of anxiety disorders	7	5.83%
Presence of depression	25	20.83%
Diabetes	45	37.50%
Arterial hypertension	102	85%
Peripheral vascular disease	22	18.33%
Vascular diseases of the CNS	12	10%
Epilepsy	3	2.50%
Head injuries	1	0.83%
Asthma	4	3.33%
Chronic obstructive pulmonary disease	7	5.83%
Smoking tobacco	53	44.17%
Anemia (Hb 10 mg/dL for female; 12 mg/dL for male)	25	20.83%
Creatinine > 1.2 mg/dL	18	15.00%
Atrial fibrillation	18	15.00%
Surgery duration (h)	3.93	0.85
ECC	91	75.83%
Intraoperative resuscitation	3	2.50%
Intraoperative steroid use	2	1.67%
Postoperative pCO_2_ ≥ 45 mmHg	25	20.83%
Postoperative pO_2_ ≤ 60 mmHg	16	13.33%
Postoperative hyperthermia	15	12.50%
Massive postoperative blood transfusion (>4 units)	6	5.00%
Plasma transfusion (≥1 unit)	15	12.50%
Length of stay in the ICU (days)	3.71	2.24
Aortic cross-clamping time (min)	36.26	28.54

CCS: Canadian Cardiovascular Society degree; CDT: Clock Drawing Test; MMSE: Mini-Mental State Examination; NYHA: New York Heart Association grade; CNS: central nervous system; pCO_2_: partial pressure of carbon dioxide; pO_2_: partial pressure of oxygen; ICU: intensive care unit; ECC: extracorporeal circulation; Hb: hemoglobin concentration.

**Table 2:** The median miRNA expression and biomarker concentrations in the whole study population.

miRNA	Pre-Operative Level Copies/mL ^a^	Post-Operative Level Copies/mL ^a^
miR-9-3p	79 (13.0–172.1)	101.4 (20.7–191.9)
miR-34c-5p	12.5 (0.0–45.4)	25.7 (0–108.1)
miR-96-5p	311.6 (110.2–696.8)	156.7 (68.7–325.7)
miR-183-5p	78.9 (14.3–179.2)	73.4 (10.4–168.2)
miR-374-3p	2.8 (0.0–30.04)	0 (0.0–16.3)
Superoxidase dismutase (ng/mL)	2.68 (2.06–3.53)	2.13 (1.62–3.01)
Antioxidant activity (µmol/L)	2.1 (1.3–2.9)	1.8 (1.2–2.6)

^a^ For continuous variables, the medians and interquartile ranges (IQRs) are given.

**Table 3:** The expression of preoperative and postoperative miRNA in univariate comparisons.

Variable	Non-Delirious ^a^	Delirious ^a^	Effect Size ^b^	*p* Value ^c^
Preoperative miR-9-3p	87.4 (34.7–187.2)	53.8 (5.1–156.6)	0.32	0.39
Preoperative miR-34c-5p	20.5 (0.0–63.1)	8.0 (0.0–42.5)	0.19	0.28
Preoperative miR-96-5p	368.2 (169.7–832.5)	165.7 (55.0–507.9)	0.49	**0.05**
Preoperative miR-183-5p	210.5 (67.7–347.6)	53.02 (9.6–173.2)	0.77	**0.0005**
Preoperative miR-374-3p	9.3 (0.0–39.7)	0 (0.0–11.2)	0.34	0.26
Postoperative miR-9-3p	118.5 (45.04–185.9)	57.13 (0–201.9)	0.32	0.39
Postoperative miR-34c-5p	51.4 (15.7–121.4)	7.6 (0–69.6)	0.56	**0.009**
Postoperative miR-96-5p	187.2 (96.5–364.2)	119.2 (33–278.6)	0.47	0.07
Postoperative miR-183-5p	89.1 (14.5–247)	39 (0–98.1)	0.49	**0.05**
Postoperative miR-374-3p	0 (0–18.0)	0 (0–15.4)	0.09	0.99

^a^ For continuous variables, the medians and interquartile ranges (IQRs) are given. ^b^ For continuous variables, Cohen’s d coefficient was calculated; for categorical variables, Cramer’s V coefficient was presented. ^c^ *p* value with Sidak correction for multiple comparisons was calculated. Significant values are in **bold**.

**Table 4:** Perioperative characteristics in univariate comparisons.

Variable	Non-Delirious ^a^	Delirious ^a^	Effect Size ^b^	*p* Value ^c^
CABG plus valve surgery	2 (3.3%)	8 (23.3%)	0.18	**0.04**
Duration of surgery (h)	4 (3–4.5)	4 (3.4–4.4)	0.15	0.41
Extracorporeal circulation	40 (66.7%)	51 (85%)	0.21	**0.02**
Intraoperative circulatory support	16 (26.7%)	17 (28.3%)	0.24	0.16
Post-op. hyperthermia > 38 °C	6 (10%)	9 (15%)	0.08	0.40
Post-op. pO_2_ ≤ 60 mmHg	5 (8.3%)	11(18.3%)	0.15	0.10
Post-op. pCO_2_ ≥ 45 mmHg	7 (11.7%)	18 (30%)	0.40	**0.01**
Plasma transfusion > 1 unit	6 (10%)	9 (15%)	0.08	0.40
Blood transfusion > 4 units	1 (6.7%)	5 (8.3%)	0.15	0.21

CABG: Coronary Artery Bypass Graft Surgery; pO_2_: partial pressure of oxygen; pCO_2_: partial pressure of carbon dioxide. ^a^ For continuous variables, the medians and interquartile ranges (IQRs) are given; for categorical variables, the number of observations (n) and fraction (%) were calculated. ^b^ For continuous variables, Cohen’s d coefficient was calculated; for categorical variables, Cramer’s V coefficient was presented. ^c^ *p* value with Sidak correction for multiple comparisons was calculated. Significant values are in **bold**.

**Table 5:** Factors independently associated with delirium after cardiac surgery revealed in a multivariate stepwise logistic regression analysis.

Variable	Coefficient	Standard Error	OR (95% CI)	*p* Value
Depression	2.53	0.79	12.6 (2.7–59.2)	<0.001
Preoperative miR-183-5p	−0.002	0.001	0.99 (0.995–0.999)	0.005
Postoperative pCO_2_ >= 45	1.32	0.62	3.7 (1.1–12.6)	0.03
Cigarette smoking	0.91	0.46	2.4 (1.007–6.15)	0.05
Female Gender	1.26	0.56	3.5 (1.2–10.5)	0.02
Peripheral vascular disease	1.38	0.68	3.9 (1.04–15.2)	0.04
Constant	−1.005	0.40	-	0.01

pCO_2_: partial pressure of carbon dioxide. The regression model is statistically significant: χ2 = 49.641, df = 6, *p* < 000.1; Hosmer–Lemeshow test: χ2 = 11.203, *p* = 0.190; Nagelkerke R^2^ = 0.451.

## Data Availability

The datasets used and/or analyzed during the current study are available from the corresponding author on reasonable request.

## Appendix A

**Table A1:** The PCR thermal cycling conditions (T100 Thermal Cycler, BioRad).

	Cycling Step	Temperature, °C	Time	Ramp Rate	No of Cycles
miR_96-5p	Enzyme activation	95	10 min	1 °C/s	1
	Enzyme activation	94	30 s		50
	Annealing/extension	62.5	1 min		50
	Enzyme deactivation	98	10 min		1
	Hold	4	Infinite		1
miR_34c-5p	Enzyme activation	95	10 min	2.5 °C/s	1
	Denaturation	94	30 s		40
	Annealing/extension	60	1 min		40
	Enzyme deactivation	98	10 min		1
	Hold	4	Infinite		1
miR_9-3p	Enzyme activation	95	10 min	1 °C/s	1
	Denaturation	94	30 s		50
	Annealing/extension	62.5	1 min		50
	Enzyme deactivation	98	10 min		1
	Hold	4	Infinite		1
miR_183-5p	Enzyme activation	95	10 min	1 °C/s	1
	Denaturation	94	30 s		50
	Annealing/extension	62	1 min		50
	Enzyme deactivation	98	10 min		1
	Hold	4	Infinite		1
miR_374-3p	Enzyme activation	95	10 min	2.5 °C/s	1
	Denaturation	94	30 s		40
	Annealing/extension	60	1 min		40
	Enzyme deactivation	98	10 min		1
	Hold	4	Infinite		1
